# Clinical profile and quality of life in late-onset and very-late-onset myasthenia gravis: a cross-sectional study

**DOI:** 10.1055/s-0046-1827053

**Published:** 2026-08-13

**Authors:** Alessandra Filpo Ferreira da Silva, Cláudia Suemi Kamoi Kay, Renata Dal-Prá Ducci, Paula Raquel do Vale Pascoal Rodrigues, Otto Jesus Hernandez Fustes, Paulo José Lorenzoni, Rosana Herminia Scola

**Affiliations:** 1 Universidade Federal do Paraná, Hospital de Clínicas, Divisão de Neurologia, Setor de Doenças Neuromusculares, Curitiba PR, Brazil.; 2 Universidade Federal do Paraná, Programa de Pós-Graduação em Medicina Interna, Curitiba PR, Brazil.

**Keywords:** Myasthenia Gravis, Quality of Life, Aged, Middle Aged

## Abstract

**Background:**

The incidence of late-onset and very-late-onset myasthenia gravis (MG) has increased worldwide, but national data in Brazil remain scarce. Characterizing the clinical profile and quality of life (QoL) of these patients is essential to improve care strategies.

**Objective:**

To describe the clinical profile and assess QoL of patients with late- and very-late-onset MG followed at a Brazilian tertiary hospital, comparing them with early-onset MG patients.

**Methods:**

Cross-sectional observational study including adult MG patients followed at a tertiary university hospital. Demographic, clinical, serological, neurophysiological, and radiological data were collected. Disease severity was assessed using the Myasthenia Gravis Composite (MGC) and Quantitative Myasthenia Gravis (QMG) scales. Furthermore, QoL was assessed using the 15-item Myasthenia Gravis QoL questionnaire (MG-QOL15). Correlation analyses were performed between MG-QOL15, MGC, and QMG scores.

**Results:**

In total, 28 MG patients were included: 13 early-onset, 7 late-onset, and 8 very-late-onset. Male predominance, shorter disease duration, and absence of thymectomy were more frequent in the late and very-late-onset groups. These subgroups also showed better symptom control and perceived QoL, particularly the very-late-onset group. The MG-QOL15 scores showed a moderate correlation with MGC and a weaker correlation with QMG.

**Conclusion:**

Late-onset and very-late-onset MG patients showed satisfactory QoL and clinical profiles, consistent with international data. These correlations reinforce the complementary role of patient-reported outcomes alongside objective clinical scales. Further studies with larger cohorts and longitudinal follow-up are needed.

## INTRODUCTION

Myasthenia gravis (MG) is a rare, chronic, autoimmune disorder that affects the neuromuscular junction, causing fluctuating muscle weakness and fatigability. It is most associated with antibodies against the acetylcholine receptor (anti-AChR), but other antibodies such as the ones against the muscle-specific tyrosine kinase (anti-MuSK) and the low-density lipoprotein receptor-related protein 4 antibody (anti-LRP4) may also be involved. Approximately 10 to 15% of patients are seronegative on routine testing. 1 2 In a Brazilian cohort, AChR antibodies were detected in 86% of patients, MuSK antibodies in 5.3%, and 8.6% were triple seronegative, with no cases positive for LRP4 antibodies, 3 highlighting the variability of antibody profiles across populations.

Although MG has historically been considered a disease predominantly affecting young women, recent decades have shown an increasing incidence in elderly populations, particularly in developed countries. 4 This demographic transition is attributed to increased life expectancy, improved diagnostic resources, and age-related immune changes favoring autoimmunity. 5

According to age at onset, MG is classified as early-onset (< 50 years), late-onset (50–64 years), or very-late-onset (≥ 65 years). 6 Studies suggest that late-onset MG has distinct clinical characteristics, often affecting men with generalized disease and positive anti-AChR antibodies.

Recent international multicenter studies have characterized these subgroups in detail. 6 7 However, Brazilian epidemiological and clinical data remain scarce. A previous study conducted in Paraná over two decades ago identified a predominance of early-onset MG patients. 8 Given the demographic changes and therapeutic advances in recent years, updated data are essential to guide care strategies for older patients with MG in Brazil. Additionally, quality of life (QoL) has become a relevant outcome in the management of chronic neuromuscular disorders such as MG, especially in elderly populations.

Therefore, the present study aimed to describe the clinical profile and assess the QoL of patients with late-onset and very-late-onset MG followed at a tertiary university hospital, comparing them with patients with early-onset MG.

## METHODS

The present was a cross-sectional and observational study conducted at the Neuromuscular Disorders Outpatient Clinic of the Complexo do Hospital de Clínicas da Universidade Federal do Paraná (CHC-UFPR), for 1 year, between August 2022 and August 2023. The local Human Research Ethics Committee approved the study, which was conducted in accordance with ethical principles (CAAE: 66636122.7.0000.009).

Adult patients (≥ 18 years) with confirmed diagnosis of MG, according to characteristic clinical manifestations associated with positive autoantibody testing and/or neurophysiological findings, were included. 1 2 All participants were under regular follow-up in the service and provided written informed consent before inclusion in the study. Patients with significant comorbidities that could potentially confound data analysis, especially severe cardiorespiratory diseases, were excluded at the investigator's discretion.

Patients were classified according to age at disease onset, into early (< 50 years), late (50–64 years), and very-late-onset MG (≥ 65 years), following previously established criteria. 6

Demographic, clinical, and ancillary test data were collected through patient interviews, physical examinations, and medical record review. Information included sex, age at disease onset, clinical presentation, comorbidities, history of thymectomy, serological tests (anti-AChR and anti-MuSK antibodies), repetitive nerve stimulation (RNS), and chest computed tomography (CT) findings.

Additionally, myasthenic crisis, previous intravenous immunoglobulin use, and therapeutic refractoriness history were evaluated. Refractory disease was defined as the persistence of disabling symptoms despite optimized treatment with at least two immunosuppressive agents at adequate doses and duration, or the occurrence of adverse effects preventing treatment continuation. 9

Disease severity was quantified using three validated instruments: the Myasthenia Gravis Composite (MGC), the Quantitative Myasthenia Gravis Score (QMG), and the 15-item Myasthenia Gravis Quality of Life Questionnaire (MG-QOL15). The MGC is a clinical scale ranging from 0 to 50 points that objectively assesses ocular, bulbar, respiratory, and limb muscle involvement. 10 11 The QMG consists of 13 items covering different muscle groups, with scores ranging from 0 to 39. It is widely used in clinical practice and research as a quantitative measure of disease severity. 12 As for MG-QOL15, it is a patient-reported questionnaire addressing physical, emotional, and social aspects of MG, with scores ranging from 0 to 60, in which higher values indicate poorer QoL. It has been culturally adapted and validated into Brazilian Portuguese 13 and has demonstrated significant correlations with clinical severity measures such as QMG and MGC. 14 15

Statistical analyses were performed using the IBM SPSS Statistics for Windows (IBM Corp.) version 29.0.2. Quantitative variables were described as means and standard deviations (SDs), while categorical variables were presented as absolute and relative frequencies. Between group comparisons for quantitative variables were conducted using one-way analysis of variance (ANOVA) or the Kruskal-Wallis test. Post-hoc pairwise comparisons were performed with Bonferroni or Dunn's tests. Categorical variables were compared using Fisher's exact test or Chi-squared test. Additionally, comparisons were made between two grouped categories: one of early-onset MG patients and the other of late-onset and very-late-onset MG patients. In these analyses, Student's *t* -test was used for normally distributed quantitative variables, and the Mann-Whitney U test for non-normally distributed ones. Correlation analyses were performed between MG-QOL15, QMG, and MGC scores using Pearson's and Spearman's coefficients, as appropriate. A significance level of *p* < 0.05 was adopted.

## RESULTS

In total, 194 patients with confirmed or suspected MG were followed at the Neuromuscular Disorders Outpatient Clinic of the CHC-UFPR in August 2022. Among those, 153 (78.8%) had early-onset MG and 41 (21.2%) had late-onset or very-late-onset MG.

**Figure 1:**
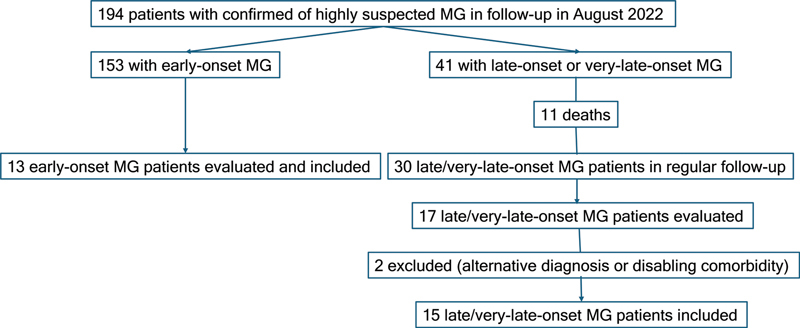
Abbreviation: MG, myasthenia gravis. Flowchart of patient selection.

A total of 28 patients were included in the final analysis: 13 with early-onset MG, 7 with late-onset MG, and 8 with very-late-onset MG. The selection process is detailed in Figure 1 .

### Demographic and clinical characteristics

Table 1 shows the demographic and clinical characteristics of patients according to age-at-onset subgroup. The proportion of male patients was significantly higher in the late-onset and very-late-onset groups compared with the early-onset group ( *p* = 0.005).

**Table 1:** Demographic and clinical characteristics according to age at disease onset

Characteristic	Early-onset ( *n* = 13)	Late-onset ( *n* = 7)	Very-late-onset ( *n* = 8)	*p* -value*
Male sex, n (%)	2 (15.4)	4 (57.1)	7 (87.5)	0.005
Generalized onset, n (%)	7 (53.8)	3 (42.9)	4 (50.0)	0.896
Age at evaluation, years (mean ± SD)	43.3 ± 10.2	62.1 ± 5.1	75.6 ± 6.0	< 0.001
Age at disease onset, years (mean ± SD)	23.8 ± 9.5	56.3 ± 2.9	70.5 ± 4.8	< 0.001

Abbreviation: SD, standard deviation.

Note: *ANOVA test (quantitative variables) and Chi-squared test (categorical variables).

Regarding the clinical presentation at disease onset, the generalized form predominated in all groups, with no significant difference between subgroups ( *p* = 0.896).

Patients' age at the time of evaluation and at disease onset was also presented in Table 1 . As expected, both the current age and the age at onset differed significantly among the three subgroups ( *p* < 0.001 for both comparisons), confirming the adequacy of age stratification in the sample. This distinction is essential to ensure analytical reliability, as overlapping ranges could compromise the validity of comparisons between groups.

All patients in the late-onset and very-late-onset subgroups reported at least one comorbidity other than MG, with hypertension and diabetes mellitus being the most frequent. In the early-onset subgroup, 70% of patients also reported additional comorbidities, most commonly hypertension, dyslipidemia, and osteoporosis.

### Ancillary tests and thymectomy history

Serological, neurophysiological, and imaging findings, as well as the history of thymectomy, are summarized in Table 2 . The results are displayed in two grouped age-at-onset categories: early-onset subgroup and late/very-late-onset subgroup.

**Table 2:** Ancillary tests and thymectomy history according to age-at-onset subgroup

Characteristic	Early-onset ( *n* = 13)	Late/very-late-onset ( *n* = 15)	*p* -value*
Anti-AChR positive, n (%)	63.6	73.3	0.683
Abnormal RNS, n (%)	84.6	93.3	0.583
Thymoma on chest CT, n (%)	15.4	0	0.333
History of thymectomy, n (%)	45.2	0	0.005

Abbreviations: AChR, acetylcholine receptor; CT, computed tomography; RNS, repetitive nerve stimulation.

Note: *Chi-squared test.

The anti-AChR antibodies were detected in most patients in all age-at-onset subgroups ( *p* = 0.683). In each subgroup, only one patient tested positive for anti-MuSK antibodies. Abnormal findings on repetitive nerve stimulation (RNS) were observed in most patients from both subgroups ( *p* = 0.583). Chest CT was performed in all patients. Thymomas were identified in two patients in the early-onset subgroup ( *p* = 0.333) and thymectomy was performed exclusively in this group ( *p* = 0.005).

### Disease severity indicators

Table 3 summarizes disease severity indicators. A minority of patients presented a history of a myasthenic crisis, with no statistically significant difference between subgroups. Alternatively, intravenous immunoglobulin (IVIg) had been used at some point in almost half of early-onset patients. Additionally, refractory disease was more frequent in early-onset MG, although without statistical significance ( *p* = 0.372).

**Table 3:** Disease severity indicators according to the age-at-onset subgroup

Characteristic	Early-onset ( *n* = 13)	Late/very-late-onset ( *n* = 15)	*p* -value*
History of myasthenic crisis, n (%)	3 (23.1)	3 (20.0)	1.000
Use of IVIg at any point, n (%)	6 (46.2)	3 (20.0)	0.228
Refractory disease, n (%)	4 (30.8)	2 (13.3)	0.372

Abbreviation: IVIg, intravenous immunoglobulin.

Note: *Chi-squared test.

### Clinical scales assessments

Table 4 summarizes the results of clinical scale assessments. The single-breath count test and the myasthenia gravis composite (MGC) score showed no statistically significant difference between subgroups. Nevertheless, this score tended to be lower in late-onset and very-late-onset groups. Similarly, the QMG score was significantly lower in older patients ( *p* = 0.024). The Dunn post-hoc analysis demonstrated the statistically significant difference between early-onset and very-late-onset patients ( *p* = 0.02).

**Table 4:** Clinical scales according to the age-at-onset subgroup

Scale/test	Early-onset ( *n* = 13)	Late-onset ( *n* = 7)	Very-late-onset ( *n* = 8)	*p* -value*
Single breath count (mean ± SD)	32.2 ± 9.6	35 ± 9.5	32.4 ± 10.6	0.769
MGC score (mean ± SD)	3.6 ± 5.0	2.9 ± 3.1	1.9 ± 2.1	0.832
QMG score (mean ± SD)	11.3 ± 4.9	10.3 ± 6.0	5.4 ± 2.3	0.024**

Abbreviations: SD, standard deviation; MGC, Myasthenia Gravis Composite; QMG, Quantitative Myasthenia Gravis.

Note: *ANOVA or Kruskal-Wallis test. ** Post-hoc Dunn test: significant difference between early-onset and very-late-onset ( *p* = 0.02).

### Quality of life assessment

The QoL evaluation, based on the MG-QOL15 score, is presented in Table 5 . Although differences did not reach statistical significance, patients with late-onset and especially very-late-onset MG showed lower mean scores than those with early-onset disease, indicating a trend toward better perceived QoL. Correlation analyses demonstrated a moderate positive association between MG-QOL15 and MGC scores, while the correlation between MG-QOL15 and QMG was weaker. As expected, a strong correlation was observed between MGC and QMG scores.

**Table 5:** Quality of life assessment according to the age-at-onset subgroup

Scale/test	Early-onset ( *n* = 13)	Late-onset ( *n* = 7)	Very-late-onset ( *n* = 8)	*p* -value*
MG-QOL15 score (mean ± SD)	8.1 ± 12.6	7.7 ± 10.6	5.6 ± 9.9	0.811

Abbreviations: MG-QOL15, 15-item Myasthenia Gravis Quality of Life Questionnaire; SD, standard deviation.

Notes: *Kruskal-Wallis test. Higher MG-QOL15 scores indicate worse quality of life.

## DISCUSSION

In this study, most patients were classified in the early-onset subgroup, in accordance with international series, such as that by Tang et al. 16 However, this finding differs from a large Spanish multicenter study, which reported a higher proportion of very-late-onset patients. 6 This discrepancy likely reflects ethnic, demographic, and referral profile differences between centers, underscoring the importance of regional studies to better characterize the Brazilian clinical scenario.

Among late-onset and very-late-onset patients, we observed a marked male predominance, consistent with several previous publications. This epidemiological pattern is already well described in European and Asian cohorts. 6 7 16

In our series, there was a similar distribution between ocular and generalized forms of disease onset in all subgroups. However, previous studies have described a higher frequency of ocular-onset MG in patients with late or very-late disease onset. In a study involving 1,160 Chinese patients, Tang et al. 16 found that the ocular subtype was more common around 50-years-old, a finding consistent with those of Živković and Lacomis 4 and Cortés-Vicente et al. 6 It is plausible that the divergence in our results is attributable to our limited sample size.

The prevalence of comorbidities was high across all subgroups, especially among late-onset and very-late-onset patients, with hypertension and diabetes mellitus being the most common. This is in line with the findings of Vijayan et al., 17 who observed a high frequency of comorbidities in very-late-onset MG patients. In that study, these comorbidities did not impact symptom severity or patient outcomes after adjustment for multiple comparisons, an aspect that deserves further exploration in Brazilian cohorts.

Regarding ancillary tests, most patients in all subgroups had positive anti-AChR antibodies and abnormal RNS suggesting postsynaptic neuromuscular junction dysfunction. The multicenter Spanish study by Cortés-Vicente et al. 6 also found no significant differences in the proportion of anti-AChR or anti-MuSK positivity between subgroups. In contrast, the recent Chinese study led by Tang et al. 16 reported a significantly higher frequency of anti-AChR positivity in late-onset and very-late-onset patients compared with early-onset ones. Interestingly, that study was the only one to compare neurophysiological findings between subgroups, identifying no significant differences—a result compatible with our own.

Chest CT revealed no thymomas among late-onset and very-late-onset patients, while previous thymectomy was significantly more frequent in early-onset patients, consistent with established therapeutic strategies. It is important to note that thymectomy, in addition to being indicated for thymomas, is a well-recognized therapeutic option in early-onset patients. 2 Recent studies, such as the one by Tang et al., 16 demonstrated that patients with thymic hyperplasia or thymoma exhibited an earlier age at onset, while those with a normal thymus were in the late-onset group. Similarly, Cortés-Vicente et al. 6 found a lower incidence of thymoma in very-late-onset MG. Older studies, however, suggested a higher incidence among elderly patients, as reported by Donaldson et al. 18 A possible explanation for this discrepancy lies in historical differences in diagnostic approaches: in past decades, broader indications for chest imaging in elderly patients with nonspecific muscle weakness, coupled with the absence of sensitive serological assays, frequently led to incidental thymoma diagnoses unrelated to MG.

In terms of disease control, including the analysis of MGC scores, frequency of myasthenic crisis, and therapeutic refractoriness—no statistically significant differences were found between subgroups, reinforcing the heterogeneity of the clinical course of MG and the influence of multiple factors beyond age at onset. The literature reports that while very-late-onset MG patients may initially present with potentially severe manifestations, they tend to respond well to treatment and have a lower likelihood of refractory disease. 6

Regarding disease severity, late-onset and very-late-onset patients exhibited lower QMG scores and MG-QOL15 scores, suggesting better clinical control and a lesser perceived impact of the disease on QoL. Although MG-QOL15 results did not reach statistical significance, possibly due to sample size limitations, there was a trend toward better perceived QoL among very-late-onset patients, especially when compared with early-onset patients.

Correlation analyses demonstrated that MG-QOL15 scores were moderately associated with MGC, and weakly associated with QMG, while MGC and QMG were strongly correlated with each other. These findings are consistent with previous studies reporting significant associations between MG-QOL15 and clinical severity measures such as QMG. 14 Nevertheless, the relationship between patient-reported outcomes and physician-reported scales is not absolute, reinforcing that QoL in MG is influenced not only by objective measures of muscle strength and clinical severity. In clinical practice, patients may present low QMG or MGC scores while still reporting relevant QoL impairment, highlighting the multidimensional nature of disease burden and the complementary role of patient-reported outcome measures in MG assessment. 19

Previous studies, such as the one by Cutter et al., 20 have shown that younger patients tend to report greater QoL impairment due to MG, possibly reflecting the higher functional impact of the disease on individuals in economically active stages of life. In our cohort, however, the early-onset subgroup presented relatively favorable MG-QOL15 scores, which may reflect specific characteristics of patients followed in a tertiary care setting, including optimized treatment, structured multidisciplinary follow-up, and longer disease duration allowing functional adaptation and coping strategies. Early therapeutic interventions, such as thymectomy and sustained immunosuppressive therapy, may also contribute to better perceived QoL over time.

This study has limitations, primarily the small sample size, which restricts the statistical power and generalizability of the results. Although early-onset MG represents the majority of patients followed at our center, the sample was kept relatively balanced across age-at-onset subgroups to preserve comparability between groups and avoid analytical bias in the analysis. Additionally, its cross-sectional design precludes assessment of long-term clinical progression and prognosis, aspects particularly relevant in age-stratified subgroups. Another limitation was the lack of systematic data regarding treatment regimens, number of immunosuppressants used, and adverse effects, which could potentially influence clinical and QoL outcomes.

In conclusion, most MG patients followed at our tertiary center were classified as early-onset. Late-onset and very-late-onset patients were characterized by male predominance, shorter disease duration, absence of thymectomy, and potentially better clinical control, as suggested by lower QMG scores. Although no statistically significant differences in QoL scores were observed, very-late-onset patients showed lower mean MG-QOL15 values, indicating a trend toward lower disease impact, which is consistent with international literature. Our findings underscore the importance of considering age-at-onset subgroups when managing MG, particularly regarding expectations for disease control and patient-reported outcomes. These results also highlight the need for national multicenter longitudinal studies, including larger patient cohorts to assess long-term outcomes, treatment response, and the role of comorbidities and immunosuppressive regimens in the QoL of Brazilian MG patients.
